# Hemodynamic Changes Following Acupuncture and Moxibustion in Patients with Subjective Tinnitus: An Acceleration Photoplethysmography and Pulsed-Wave Doppler Ultrasound Study

**DOI:** 10.3390/audiolres16050130

**Published:** 2026-09-02

**Authors:** Ji-Won Lee, Seung-Ug Hong, Sung-Yun Park

**Affiliations:** 1 Department of Ophthalmology, Otolaryngology and Dermatology, College of Korean Medicine, Dongguk University, Goyang 10326, Republic of Korea; behelia11@gmail.com (J.-W.L.); heenthsu@duih.org (S.-U.H.); 2 Department of Diagnostics, College of Korean Medicine, Dongguk University, Goyang 10326, Republic of Korea

**Keywords:** acupuncture, moxibustion, photoplethysmogram, tinnitus, pulsed-wave Doppler ultrasound

## Abstract

**Background/Objectives:** Tinnitus has been linked to hemodynamic changes. Objective measures capable of monitoring hemodynamic changes during tinnitus treatment remain limited. This study investigated hemodynamic changes during combined acupuncture and moxibustion treatment in patients with subjective tinnitus using photoplethysmography (PPG) and pulsed-wave Doppler ultrasound (PW Doppler). **Methods:** A retrospective review of 10 patients with subjective tinnitus treated with acupuncture and moxibustion was conducted. Hemodynamic changes were assessed using acceleration photoplethysmogram (APG) indices and PW Doppler parameters. **Results:** The c/a ratio in APG increased significantly after acupuncture, indicating an acute change in arterial pulse wave characteristics.. Combined acupuncture and moxibustion significantly increased peak systolic velocity (PSV), end-diastolic velocity (EDV), and mean flow velocity (MV), while decreasing the resistive index (RI) in the superficial temporal artery. However, improvements in the Tinnitus Handicap Inventory (THI) scores did not consistently align with changes in APG and PW Doppler parameters. **Conclusions:** These findings suggest that APG and PW Doppler may be useful for objectively assessing acute hemodynamic changes during acupuncture and moxibustion treatment in patients with subjective tinnitus.

## 1. Introduction

Tinnitus, the perception of sound without an external source, affects a significant number of individuals and often leads to chronic discomfort and reduced quality of life [1,2]. While the underlying mechanisms of tinnitus are complex and multifactorial, growing evidence suggests that cochlear dysfunction plays an important role in its pathophysiology [2,3]. The inner ear, particularly the hair cells, is highly sensitive to blood flow, and its function can be compromised by hypoxia resulting from microcirculatory disturbances [4]. This has led to increasing interest in the role of vascular factors in tinnitus. Studies have reported associations between tinnitus and conditions such as cardiovascular disease, dyslipidemia, and diabetes [5,6,7]. These conditions may impair cochlear microcirculation and reduce blood supply to the inner ear [4]. These findings suggest that vascular regulation may contribute to tinnitus symptoms in at least some patients. However, objective measures that can capture hemodynamic changes during tinnitus treatment remain limited [8,9,10].

Addressing this limitation requires an intervention that elicits measurable hemodynamic responses. Pharmacological treatments, including vasodilators, atorvastatin, and Ginkgo biloba extract, are widely used to improve cochlear blood flow in patients with tinnitus, although their clinical benefits remain limited, particularly in chronic or severe cases [11,12]. Acupuncture and moxibustion, key components of traditional Asian medicine, are widely used as complementary treatment options for patients whose symptoms remain inadequately controlled with pharmacological therapy [13,14,15,16,17]. Acupuncture may modulate vascular and autonomic responses, while moxibustion provides additional thermal stimulation that may promote local blood flow [14,16,18,19,20]. Unlike conventional symptom-based assessments, the hemodynamic responses induced by acupuncture and moxibustion can be repeatedly assessed while treatment is being delivered without interrupting the therapeutic process. This enables objective evaluation of hemodynamic changes during treatment rather than relying solely on post-treatment clinical outcomes. In routine clinical practice, acupuncture and moxibustion are commonly administered together over repeated treatment sessions. However, despite increasing research on the individual effects of acupuncture and moxibustion [13,14,16,18,19,20], few studies have objectively characterized the physiological responses that occur during their combined application in patients with tinnitus.

Objective assessment of hemodynamic changes during acupuncture and moxibustion treatment requires non-invasive techniques that permit repeated measurements without interrupting the therapeutic process. Although invasive techniques provide accurate hemodynamic measurements, they may interfere with treatment and alter the physiological responses being evaluated, making it difficult to distinguish genuine physiological responses from those influenced by the examination itself [21]. Several non-invasive imaging techniques, including functional near-infrared spectroscopy (fNIRS) [22], functional magnetic resonance imaging (fMRI) [23], and Technetium-99m Ethyl cysteinate dimer single photon emission computed tomography (Tc-ECD SPECT) [24,25], have been used to evaluate cerebral blood flow in patients with tinnitus. However, these techniques primarily assess cortical hemodynamics and are less suitable for repeated monitoring of hemodynamic changes during treatment.

Among the available non-invasive techniques, photoplethysmography (PPG) and ultrasound (US) are particularly well-suited for repeated assessment of hemodynamic changes during treatment [26,27,28,29,30]. The acceleration photoplethysmogram (APG), the second derivative of PPG, provides quantitative assessment of arterial pulse wave characteristics [28]. Pulsed-wave (PW) Doppler US enables real-time measurement of regional blood flow without interrupting the therapeutic process [30]. Together, APG and PW Doppler provide complementary assessment of systemic pulse wave characteristics and regional blood flow, enabling objective monitoring of hemodynamic changes during treatment. However, their application for monitoring hemodynamic changes during tinnitus treatment remains limited [28]. Doppler studies in tinnitus have mainly focused on the carotid artery, with little attention to temporal artery blood flow during treatment [31,32].

To address this gap, we investigated hemodynamic changes during combined acupuncture and moxibustion treatment in patients with subjective tinnitus using APG and PW Doppler US. APG was used to evaluate changes in arterial pulse wave characteristics, and PW Doppler US was used to assess blood flow in the temporal artery. We also analyzed APG and PW Doppler parameters in individual patients to examine variability in hemodynamic responses during treatment.

## 2. Materials and Methods

### 2.1. Study Protocol

A retrospective chart review was conducted on patients with subjective tinnitus who visited Dongguk University Ilsan Oriental Hospital between April and July 2024. APG and PW Doppler measurements were performed as part of routine clinical assessment, and the resulting data were retrospectively reviewed for this study. The research adhered to the ethical principles outlined in the Declaration of Helsinki for studies involving human participants. Ethical approval for this study was obtained from the hospital’s Institutional Review Board (IRB protocol number: DUIOH 2024-06-006-001). As patient data were collected from existing medical records, the IRB granted a waiver for informed consent. All patients included in the study underwent standard diagnostic and treatment procedures. This research followed established reporting standards for observational studies, including the STROBE guidelines, with a completed checklist provided as Supplementary Material.

### 2.2. Study Designs

A retrospective chart review was performed on patients with subjective tinnitus who sought care at the Ophthalmology, Otolaryngology, and Dermatology outpatient department at Dongguk University Ilsan Oriental Hospital in Goyang, Republic of Korea, between April and July 2024. The study included only those patients for whom APG and PW Doppler data were available. The data extracted from the electronic medical records included patient demographics, detailed medical history, medication usage, tinnitus diagnosis and characteristics, APG and PW data, as well as treatment specifics and their outcomes. The causes of tinnitus were identified from physician-documented diagnoses and clinical history in the medical records.

### 2.3. Participants

#### 2.3.1. Inclusion Criteria

The inclusion criteria were:

1. Outpatients with a documented clinical diagnosis of subjective, non-pulsatile tinnitus (unilateral or bilateral);
2. Patients with available APG and PW Doppler US data.

#### 2.3.2. Exclusion Criteria

The exclusion criteria were [33]:

1. The presence of diseases known to affect hemodynamic status, including cardiovascular, endocrine, respiratory, autoimmune, neurologic, and psychiatric disorders, as well as conditions such as alcoholism and polytoxemia;
2. A history of smoking;
3. Documented use of vasoconstrictors, beta-blockers, anticholinergics, antidepressants, or other central nervous system-active medications within the past four weeks;
4. Pregnancy.

### 2.4. PPG and PW Doppler US Measurements

**Figure 1:**
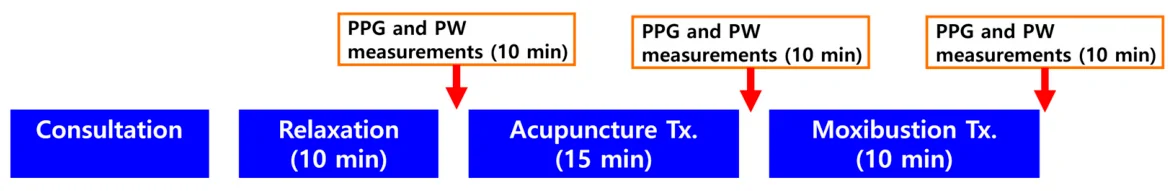
Outpatient Treatment Protocol for Managing Subjective Tinnitus.

The standard outpatient treatment protocol for tinnitus is presented (Figure 1). PPG and PW Doppler US measurements were performed at each outpatient treatment session in which acupuncture and moxibustion were administered. Measurements were conducted three times during each session: prior to treatment, after acupuncture, and after moxibustion. All measurements were conducted in a controlled environment with minimal air movement, no direct sunlight or other radiation sources, and clinical temperature and humidity levels maintained according to the manufacturer’s guidelines.

#### 2.4.1. PPG Measurements

**Figure 2:**
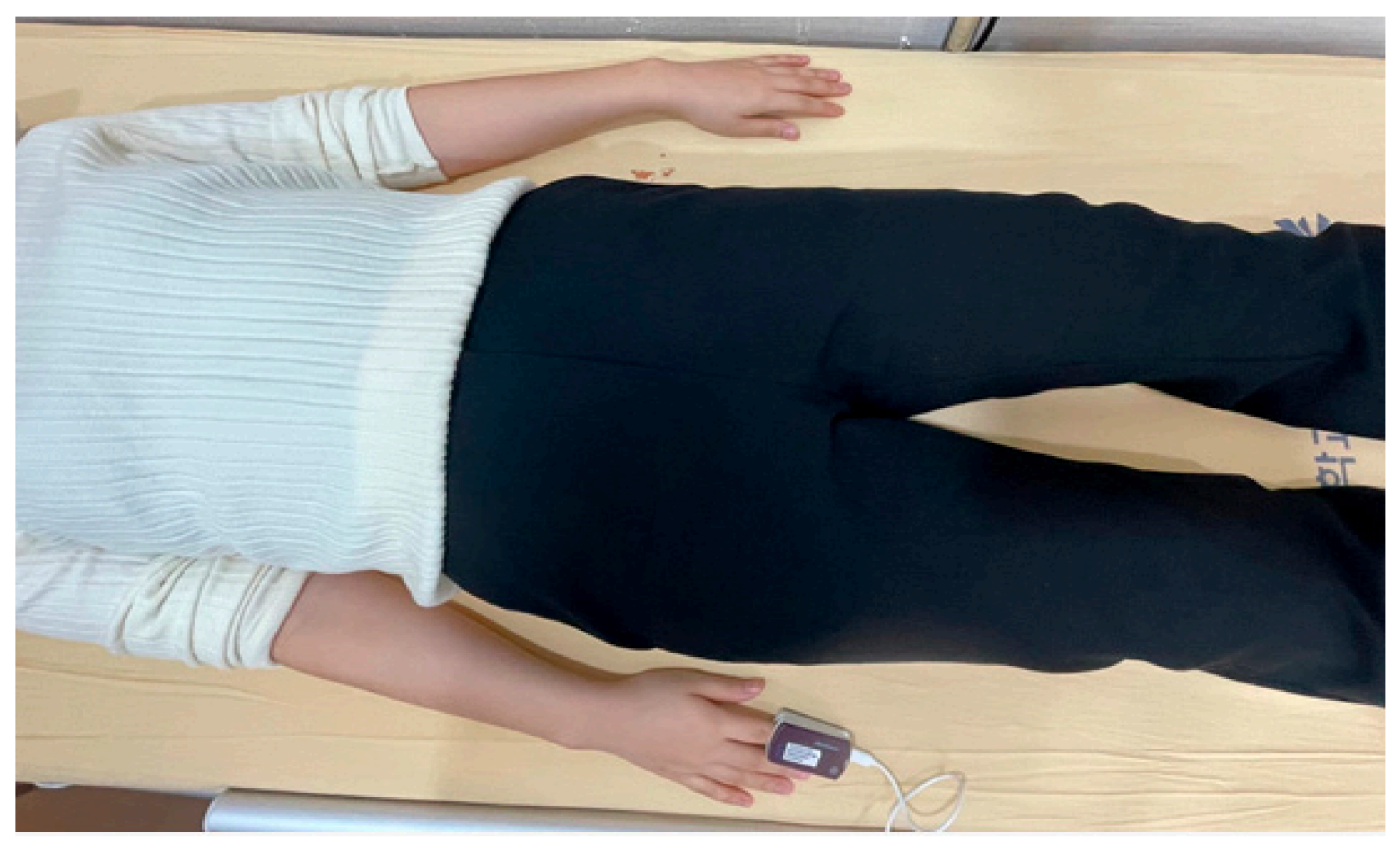
Procedure for Photoplethysmogram Measurements in Tinnitus Patients.

Following an initial consultation, patients were instructed to remove any metal objects and lie on comfortable beds within a quiet, temperature-controlled room. Following a 10-min relaxation period to stabilize breathing and heart rate, participants remained in a supine position while PPG data were collected for 5 min using the uBioMacpa device (Biosense Creative Inc., Seoul, Republic of Korea) (Figure 2). The collected data were subsequently analyzed.

#### 2.4.2. PW Doppler US Measurements

**Figure 3:**
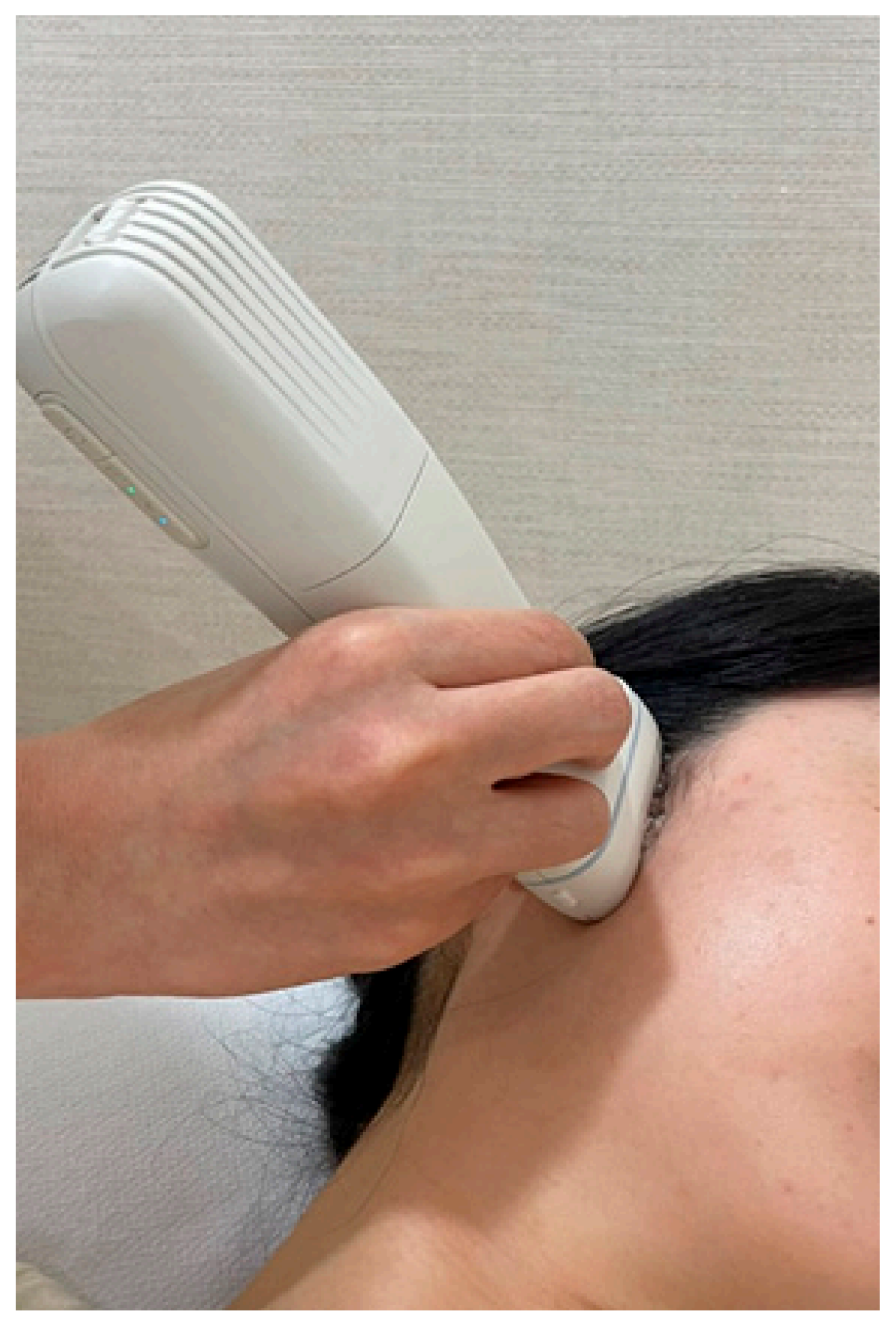
Pulsed-Wave Doppler Ultrasound Measurement Procedure in Tinnitus Patients.

As the Tinggong (SI19) was the primary acupoint for both acupuncture and moxibustion treatments [34], it was chosen as the site for Doppler US measurements. The superficial temporal artery near SI19 was selected as an accessible peripheral vessel but does not directly represent cochlear blood flow. For unilateral tinnitus, PW Doppler measurements were performed on the affected side. For bilateral tinnitus, both sides were measured independently. Each participant underwent a PW Doppler US evaluation using the Healcerion SONON 500L device (Healcerion, Seoul, Republic of Korea) with a linear probe. A sufficient amount of water-soluble gel was applied to the affected aural area and the US transducer head. Once the temporal artery near the SI19 was located, anatomical marks and US sites were identified using a hypoallergenic surgical marking pen. An experienced sonographer with registered in Musculoskeletal (RMSK) sonography certification by Alliance for Physician Certification & Advancement (APCA), conducted all US measurements. The US measurements were performed with the participants lying comfortably on a bed in the supine position with their arms in supination (Figure 3). PSV, EDV, RI, and MV were selected to assess arterial flow velocity and vascular resistance during treatment. Other Doppler indices were not routinely recorded and therefore were not included in the retrospective analysis.

### 2.5. Interventions

In our outpatient tinnitus treatment, acupuncture is usually performed first, followed by moxibustion. Throughout both procedures, participants were closely monitored for any signs of discomfort. All treatment sessions were conducted in the same room as the PPG and PW Doppler US measurements, in a controlled environment with consistent temperature, no air movement, and no exposure to direct sunlight or other radiation sources.

#### 2.5.1. Acupuncture Treatment

**Figure 4:**
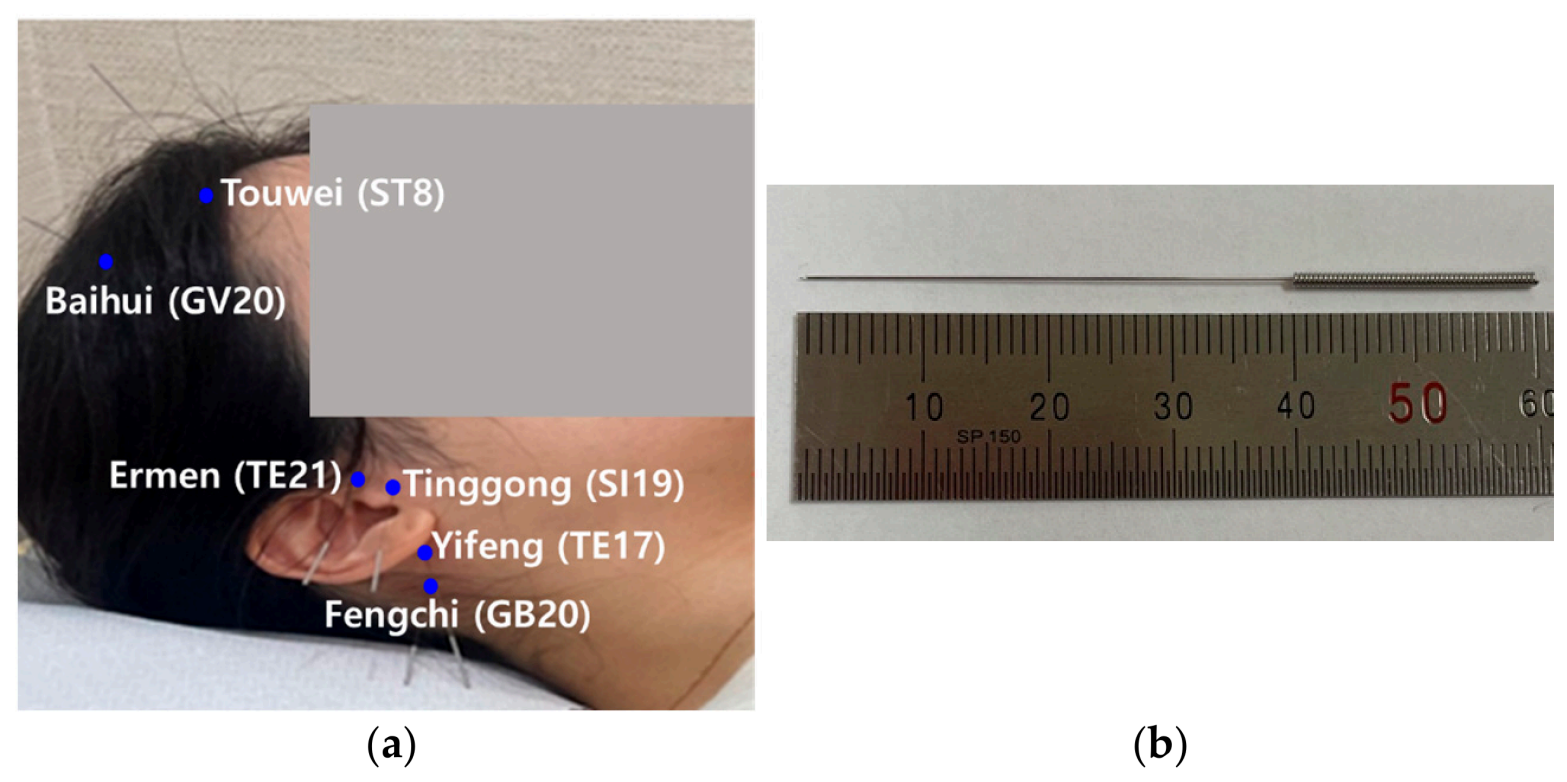
(**a**) Acupuncture treatment procedure. (**b**) Standardized disposable stainless steel needles (0.25 × 40 mm, Dongbang Medical, Boryeong, Republic of Korea).

A licensed Korean medicine doctor with over three years of experience performed acupuncture using standardized disposable stainless steel needles (0.25 × 40 mm, Dongbang Medical, Boryeong, Republic of Korea). Participants lay comfortably on a bed in a supine position during the acupuncture procedure. Acupuncture was applied to specific points, including Baihui (GV20), Yifeng (TE17), Ermen (TE21), Fengchi (GB20), and Tinggong (SI19) on the affected side, as well as Touwei (ST8) and Hegu (LI4) on both sides (Figure 4). After sterilizing their hands and the acupoints where the needles were to be inserted, the practitioner employed the nail-pressing method for needle insertion, adjusting the technique based on the characteristics of each acupoint. The direction and depth of insertion followed strict acupuncture manipulation protocols. After insertion, twisting, lifting, thrusting, and the reducing method were applied until a ‘Deqi’ sensation was achieved, with lifting and thrusting between 0.3 and 0.5 cm, and twisting around 180° at a frequency of 50–60 cycles per minute. The needles remained in place for 15 min without further manipulation. During removal, the practitioner used a sterilized dry cotton ball held by the left thumb and index finger to gently press the acupuncture points, while the right hand carefully twisted and lifted the needles to the subcutaneous layer, paused briefly, and then slowly withdrew them. The pinholes were not pressed after removal unless bleeding occurred.

#### 2.5.2. Moxibustion Treatment

**Figure 5:**
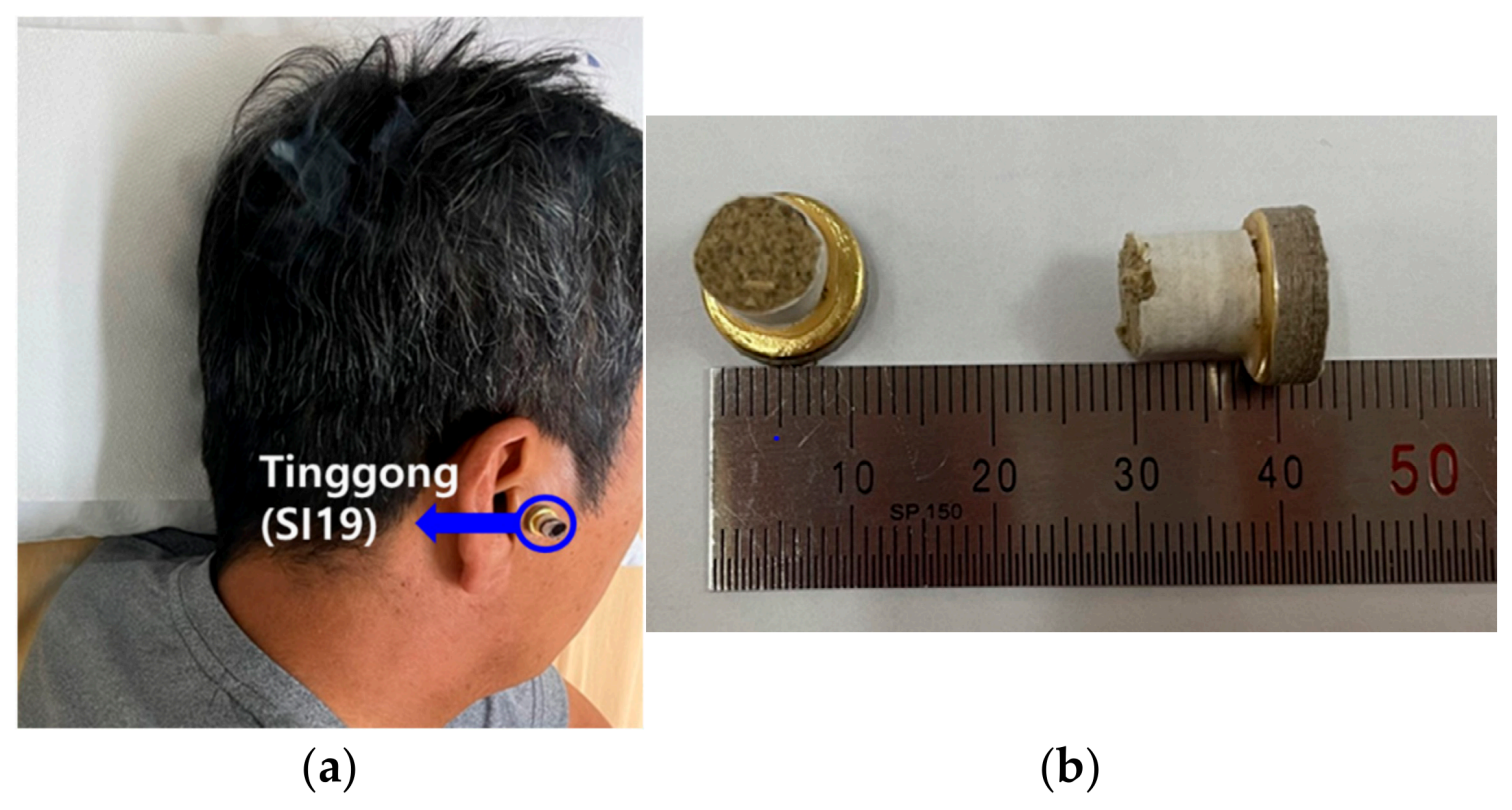
(**a**) Moxibustion treatment procedure. (**b**) Moxa rolls (Dongbang Medical, Boryeong, Republic of Korea).

After acupuncture, the same practitioner administered moxibustion to the SI19 point on the affected side, with the skin at the acupoint sterilized beforehand. The patient was positioned laterally with the affected ear facing upward, and non-contact moxibustion was applied using a moxa roll made from dried mugwort leaves in a paper cylinder (Dongbang Medical, Boryeong, Republic of Korea). The lit moxa roll was held approximately 1 cm above the skin at the acupoint (Figure 5). The procedure was repeated three times, lasting a total of 10 min. A gentle, warm sensation, similar to the ‘Deqi’ sensation experienced during acupuncture, was achieved before any redness appeared on the skin.

### 2.6. Data Assessment

#### 2.6.1. Sample Characteristics

The following descriptive variables were extracted from participants’ electronic medical records:

1. Gender;
2. Age;
3. Tinnitus-related variables: onset, tinnitus location (bilateral or unilateral), cause of onset (e.g., stress, trauma, sudden hearing loss, otitis media, Meniere’s disease), Tinnitus Handicap Inventory (THI) score at the first visit, and ear-related symptoms (e.g., aural fullness, hearing loss, otalgia, vertigo);
4. Treatment-related variables: number of outpatient treatments. (a) Moxibustion treatment procedure and (b) Moxa rolls (Dongbang Medical, Dongbang Moxa).

#### 2.6.2. Outcome Variables

**Figure 6:**
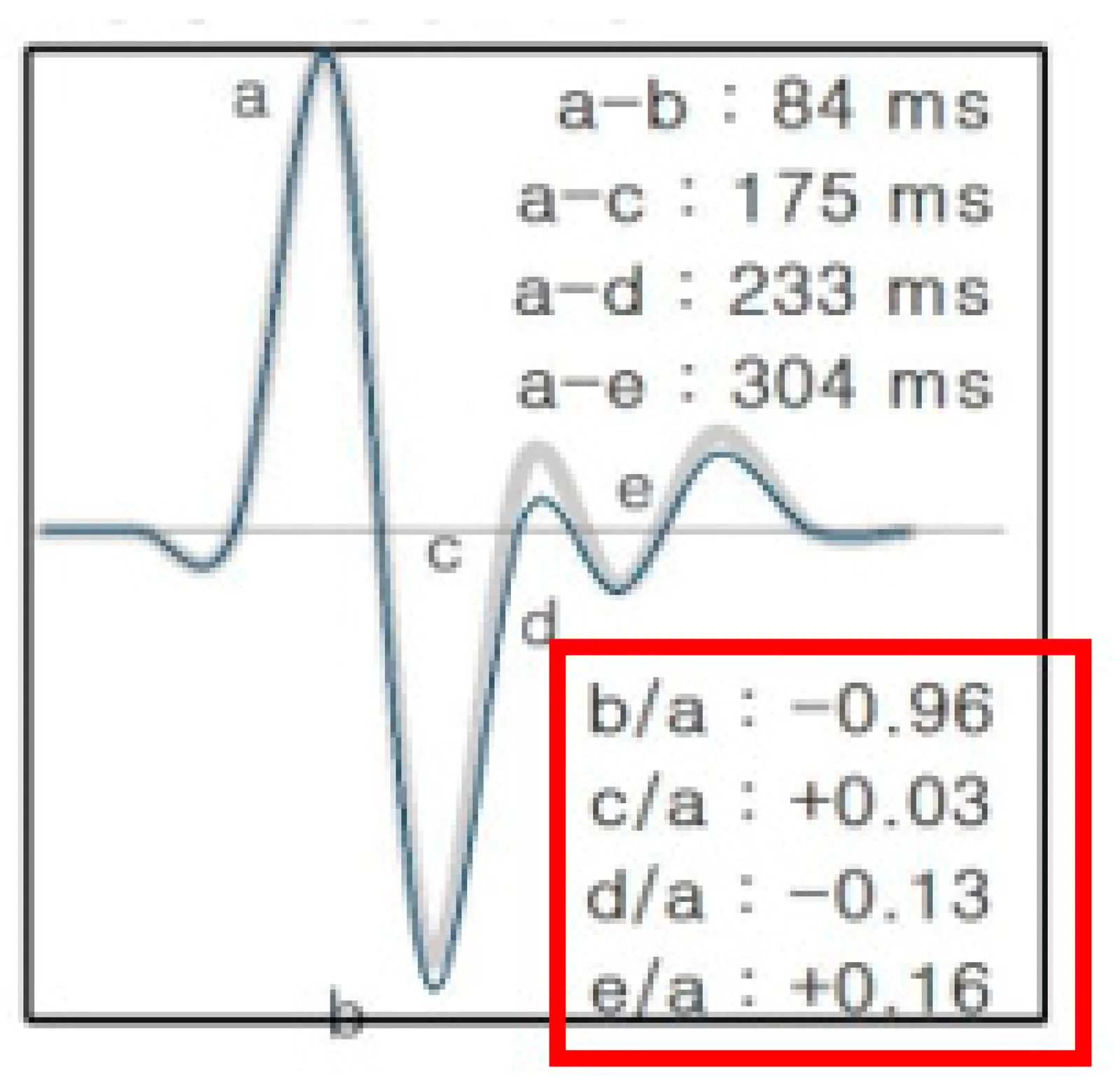
Photoplethysmogram Second Derivative Waveform and Acceleration Photoplethysmogram Variables. The labels a–e indicate the a-, b-, c-, d-, and e-waves of the APG waveform, respectively.

**Figure 7:**
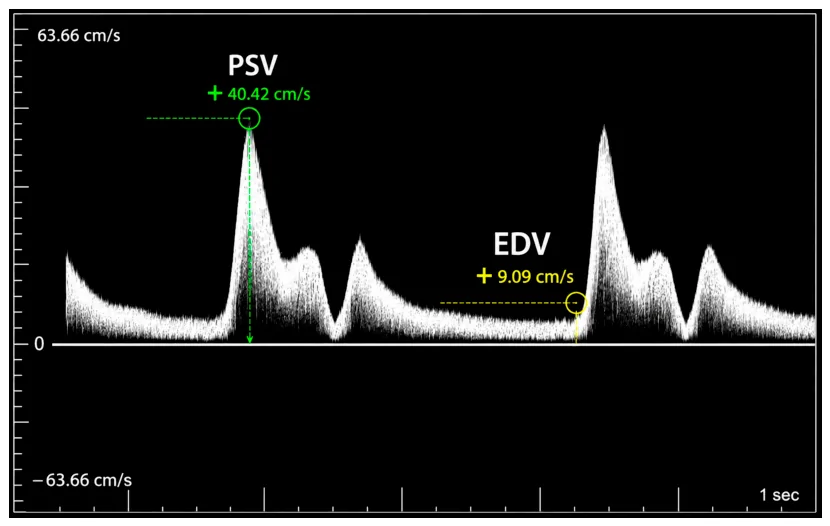
Analyzed Pulsed-Wave Doppler Ultrasound Image of Temporal Artery.

1. APG variables: The APG waveform consists of four systolic waves and one diastolic wave, identified as the a-wave (early systolic positive wave), b-wave (early systolic negative wave), c-wave (late systolic re-increasing wave), d-wave (late systolic re-decreasing wave), and e-wave (early diastolic positive wave). The height of each wave was measured from the baseline, with positive values above the baseline and negative values below it. For wave analysis, the ratios of wave heights to the a-wave (b/a, c/a, d/a, e/a) were calculated (Figure 6). All APG variables were analyzed using the software of the uBioMacpa device [26].

2. PW Doppler US variables: Based on prior research and standard PW Doppler measurements [30], the following variables were analyzed: Peak Systolic Velocity (PSV), End Diastolic Velocity (EDV), Resistive Index (RI), and Mean Flow Velocity (MV) (Figure 7). The variables for each Doppler waveform were calculated using the built-in software of the US system or Python 3.13 (The Python Software Foundation, Wilmington, DE, USA).

### 2.7. Statistical Analysis

Statistical analyses were performed using Python 3.13 (The Python Software Foundation, Wilmington, DE, USA), with statistical significance set at *p*-value < 0.05. Continuous variables were expressed as means with standard deviations (SD) or standard errors (SE), while categorical variables were summarized as counts and percentages. To assess the effects of treatment on APG and PW parameters, Linear Mixed-Effects Models (LMMs) were employed, treating the intervention as a fixed effect and patients as random effects. Repeated measurements across multiple outpatient visits were accounted for by including patients as random effects in the LMMs. Visit frequency and treatment duration were not included as covariates. The normality of residuals from the LMMs was evaluated using the Shapiro–Wilk test, with normality defined by a *p*-value > 0.05 or a statistical threshold exceeding 0.9. Individual analyses were limited to patients with at least three treatment sessions to ensure sufficient repeated measurements for paired comparisons. For these patients, repeated pre- and post-treatment measurements across multiple treatment sessions were analyzed. Pre- and post-treatment differences were subjected to the Shapiro–Wilk test for normality. Based on the results of the normality test, either a paired *t*-test or Wilcoxon signed-rank test was subsequently applied to assess whether the mean of these differences was statistically significantly different from zero.

## 3. Results

### 3.1. Participant and Practice Characteristics

**Figure 8:**
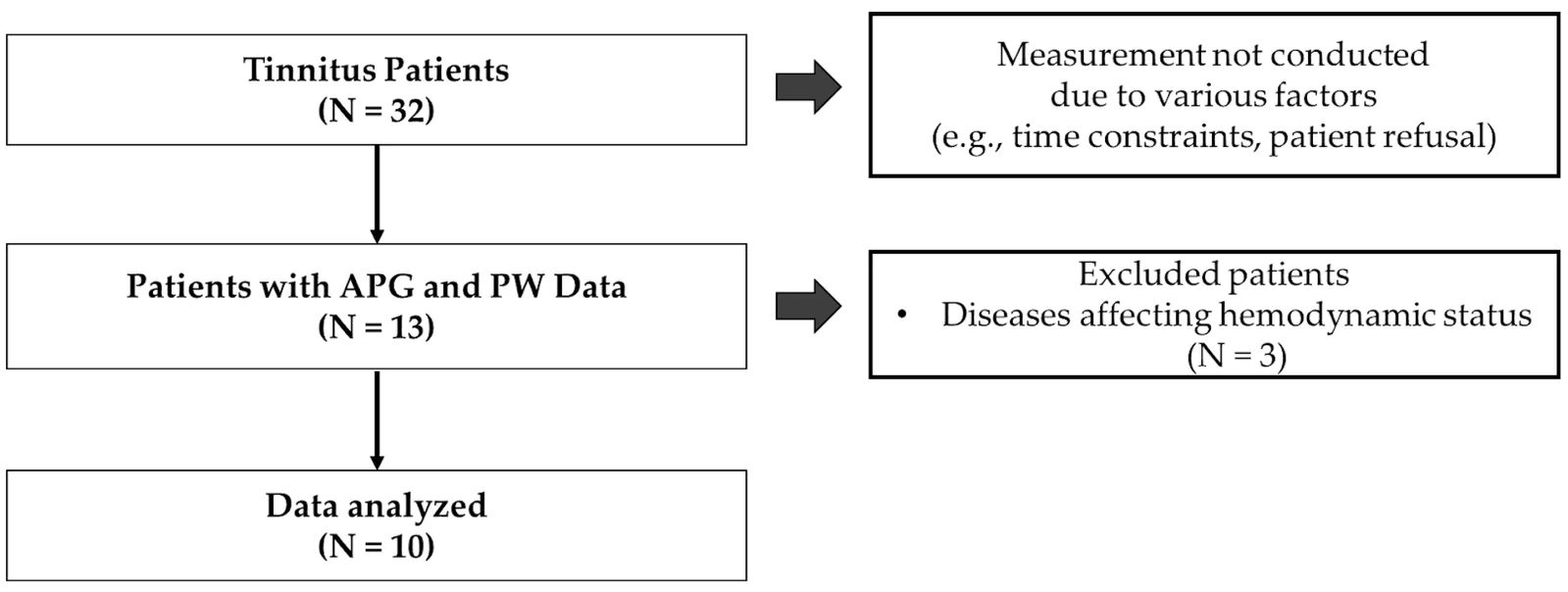
Flowchart Depicting Retrospective Chart Review Process for Tinnitus Patients.

A flow diagram of the retrospective chart review process is presented (Figure 8), which resulted in the analysis of 10 tinnitus patients. The characteristics of these patients are summarized (Table 1). The mean age of the patients was 41.8 years (±18.4). Sixty percent (*n* = 6) were male, and the average duration of tinnitus symptoms was 12.1 months (±11.5). Most patients (80%, *n* = 8) had unilateral tinnitus, while 20% (*n* = 2) had bilateral tinnitus. The causes of tinnitus varied, with 40% (*n* = 4) having an unknown cause. Other causes included stress (10%, *n* = 1), trauma (10%, *n* = 1), sudden hearing loss (20%, *n* = 2), otitis media, Meniere’s disease, or flu (10%, *n* = 1), and benign paroxysmal positional vertigo (BPPV) (10%, *n* = 1). The mean number of outpatient treatments was 6.9 (±3.9), and the average pre-treatment THI score was 26.8 (±12.9). Common symptoms included aural fullness (70%, *n* = 7), hearing loss (40%, *n* = 4), otalgia (40%, *n* = 4), and vertigo (20%, *n* = 2).

### 3.2. Changes in APG Parameters Before and After Treatment

In this study, LMMs were applied to account for individual variability among patients while assessing changes in APG parameters across three groups: Base (before treatment), AT (after acupuncture), and AT + MT (after acupuncture combined with moxibustion). The mean values and SD for the ‘b/a’, ‘c/a’, ‘d/a’, and ‘e/a’ ratios are presented (Table 2).

**Figure 9:**
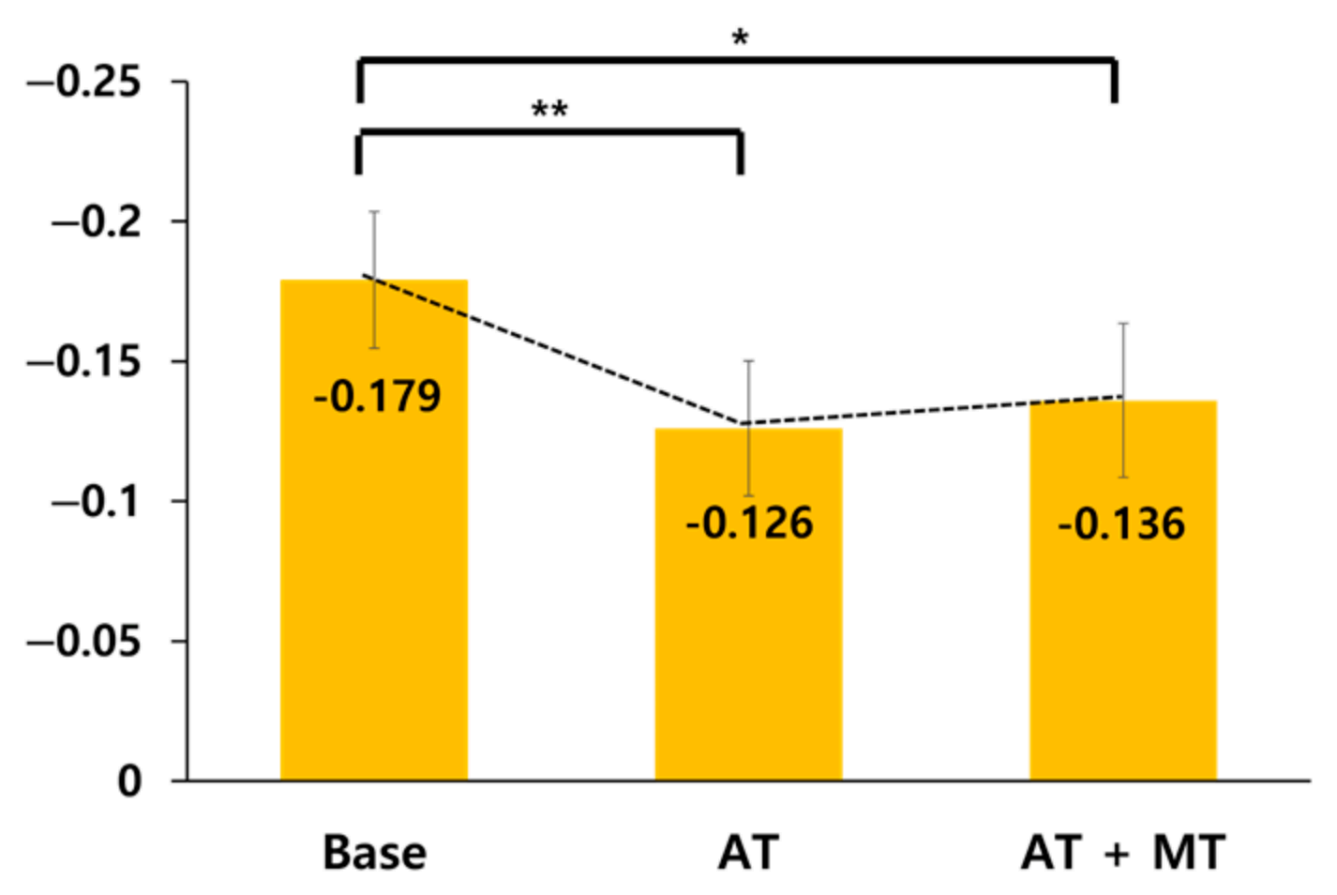
Mean ‘c/a’ Ratio and Standard Error Bars Across Pre-Treatment, Post-Acupuncture, and Combined Acupuncture with Moxibustion Groups. **, *p*-value < 0.01; *, *p*-value < 0.05.

The analysis revealed significant differences in the ‘c/a’ ratio for both the Base vs. AT (Effect = 0.053, *p* = 0.002) and Base vs. AT + MT (Effect = 0.043, *p* = 0.020) comparisons (Table 3). In contrast, the ‘b/a’ ratio did not show statistically significant changes, with *p*-values ranging from 0.425 to 0.739. The mean values with SE bars for the c/a ratio across the three groups, as well as the changes in the c/a ratio before and after acupuncture and moxibustion, are illustrated (Figure 9).

There were no significant differences in the ‘d/a’ and ‘e/a’ ratios across all comparisons, as *p*-values were all above 0.05 (Table 4). Additionally, there were no significant changes when comparing AT and AT + MT across any of the indicators. Overall, group variance remained low throughout most comparisons, particularly for the ‘e/a’ ratio, which showed very minimal variability.

### 3.3. Changes in PW Doppler US Parameters Before and After Treatment

The mean values and SD for PSV, EDV, RI, and MV across the Base, AT, and AT + MT groups are summarized (Table 5). LMMs were applied to account for individual variability.

The comparison between Base and AT showed no statistically significant changes in PSV or EDV (Table 6). However, the comparison between Base and AT + MT revealed significant increases in both PSV (Effect = 4.36, *p* = 0.020) and EDV (Effect = 1.807, *p* = 0.001). No significant changes in PSV or EDV were observed between AT and AT + MT.

**Figure 10:**
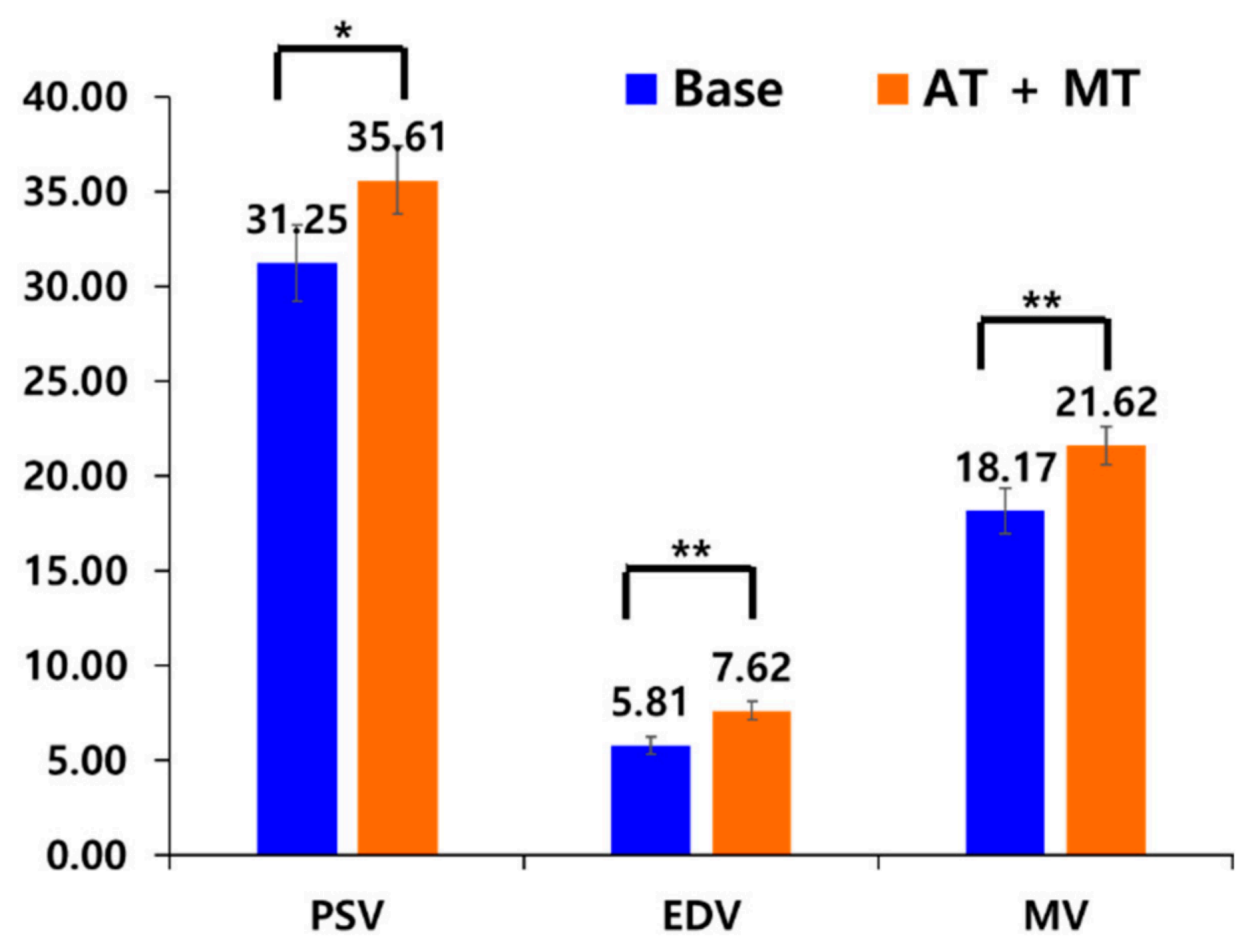
Comparison of Mean Values and Standard Errors for PW Doppler Parameters in Base and AT + MT Groups. **, *p*-value < 0.01; *, *p*-value < 0.05.

No significant differences were found between Base and AT for either RI or MV (Table 7). However, when comparing Base to AT + MT, significant decreases in MV (Effect = 3.451, *p* = 0.002) and a minor decrease in RI (Effect = −0.027, *p* = 0.014) were noted. No significant changes in RI or MV were observed between AT and AT + MT. Group variance was substantial for several parameters, particularly for PSV and MV, indicating notable variability across patients. The mean values and SE bars for the Base and AT + MT groups are presented (Figure 10).

### 3.4. APG and PW Doppler US Parameters Pre- and Post-Treatment Differences Among Subjects

**Figure 11:**
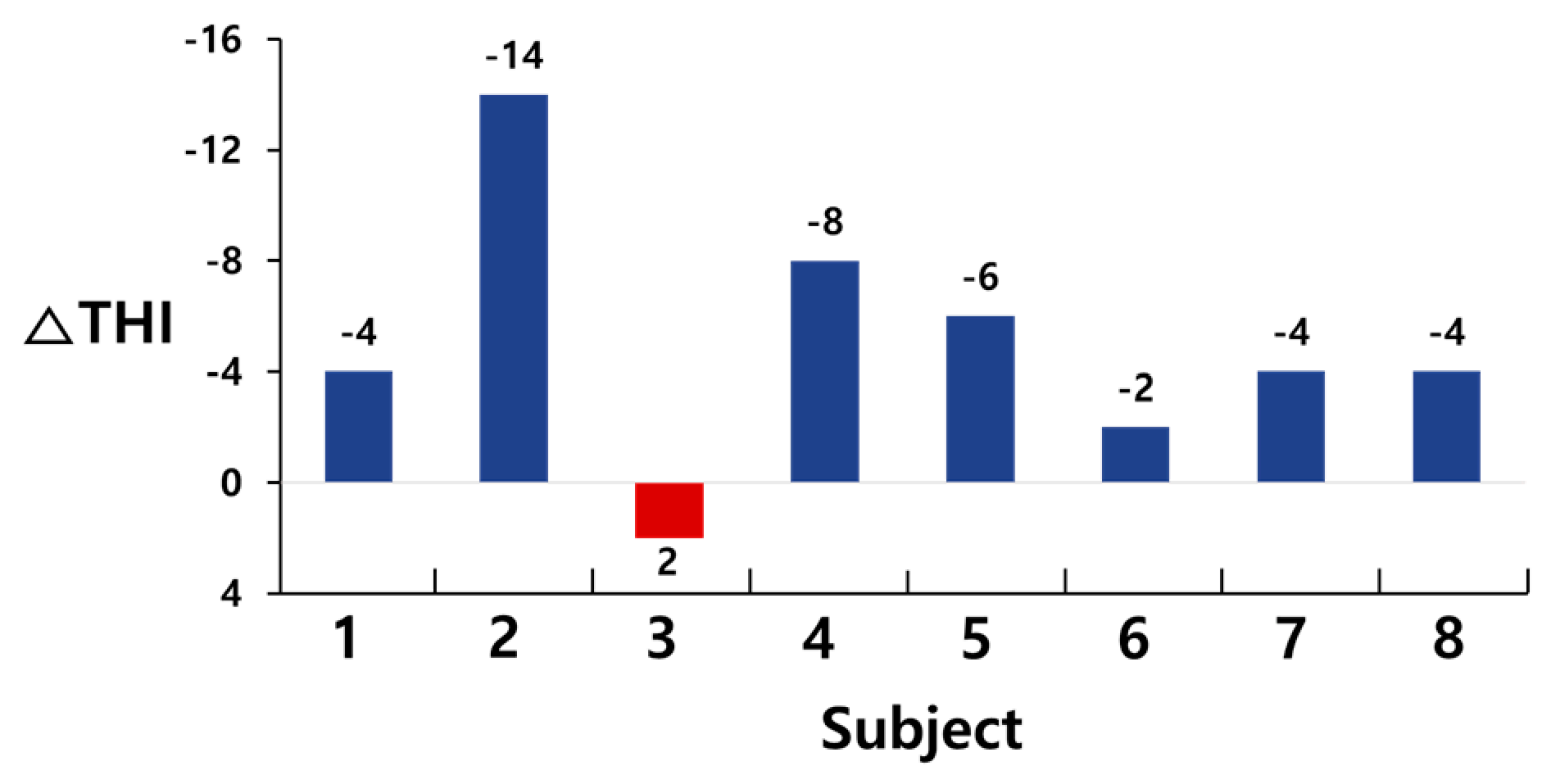
Tinnitus Handicap Inventory (THI) Scores: Changes Between the Last and First Outpatient Visits for Individual Patients. Blue columns indicate negative changes in THI scores, whereas red columns indicate positive changes.

The changes (Δ) in THI scores, APG metrics (Δb/a, Δc/a, Δd/a, Δe/a), and PW Doppler parameters (ΔPSV, ΔEDV, ΔRI, ΔMV) for eight subjects with at least three outpatient visits are presented (Table 8). Subject 2, who showed the greatest improvement in THI (−14), displayed moderate increases in ΔPSV, ΔEDV, ΔRI, and ΔMV, though none were statistically significant. This suggests that, even without significant changes in the APG and PW Doppler metrics, a substantial subjective improvement in tinnitus was reported. Subject 3 showed significant improvements in Δb/a (*p* = 0.035), Δd/a (*p* = 0.001), ΔPSV (*p* = 0.045), ΔEDV (*p* = 0.004), and ΔMV (*p* = 0.018), despite a slight increase in THI (+2). On the other hand, Subject 4 exhibited significant changes in Δc/a (*p* = 0.048) and ΔRI (*p* = 0.035), along with a noticeable reduction in THI (−8), suggesting that these physiological changes may be linked to improvements in tinnitus severity. Similarly, Subject 8 showed a significant change in Δc/a (*p* = 0.043) and a moderate decrease in THI (−4). Overall, these results suggest that THI improvements may not always align with significant changes in APG and PW Doppler parameters. The changes in THI scores for individual patients between their first and last outpatient visits are shown (Figure 11).

## 4. Discussion

This study examined hemodynamic changes during acupuncture and moxibustion treatment in patients with subjective tinnitus using APG and PW Doppler US.

### 4.1. Participant Characteristics and Their Clinical Implications

The study’s cohort consisted predominantly of middle-aged patients with unilateral tinnitus and varying etiologies, including cases of unknown origin, sudden hearing loss, and stress-related causes. The diversity in symptom duration, underlying causes, and accompanying symptoms—such as aural fullness, hearing loss, and otalgia—underscores the multifactorial nature of tinnitus and the need to tailor acupuncture and moxibustion treatments based on patient-specific factors [3,35]. The moderate pre-treatment THI scores indicate the potential that patients may be responsive to therapeutic interventions [36].

### 4.2. Hemodynamic Changes Observed in APG Parameters

PPG sensors can be placed on any superficial vessel and allow for immediate physiological measurements [26,28]. PPG can provide heart rate from the cyclical variation in blood volume over time and additional information, such as pulse rate variability [28]. The second derivative of PPG, known as APG, provides more detailed cardiovascular insights than the first derivative [28]. APG indices, such as b/a, c/a, d/a, and e/a, are used to assess vascular stiffness. Specifically, the b/a index increases with arterial stiffness, while the c/a, d/a, and e/a indices decrease. While a number of indices have been reported to indicate vascular stiffness, it remains unclear which of these indices is most informative [26,37].

The present findings showed that the c/a ratio increased after acupuncture, indicating a measurable change in arterial pulse wave characteristics during treatment [26,37]. An increased c/a ratio has been associated with changes in vascular tone or compliance, suggesting that the observed hemodynamic response may reflect an acute vascular response during acupuncture treatment. This finding aligns with previous research showing a notable reduction in the APG-d/a index following acupuncture at the Pericardium 6 (PC6) acupoint, indicating a decrease in left ventricular afterload [38]. Acupuncture is thought to regulate the release of vasoactive substances such as nitric oxide (NO), which induces vasodilation by relaxing vascular smooth muscles, and endothelin-1 (ET-1), a potent vasoconstrictor [39].

The absence of significant changes in other APG indices (e.g., ‘b/a’, ‘d/a’, ‘e/a’) could be attributed to the relatively short duration of intervention or the specific acupoints chosen [38,40]. Additionally, the sensitivity of PPG sensors to factors such as motion artifacts, ambient light conditions, contact pressure, sensor placement, body location, body temperature, and skin color can impact measurement accuracy [28]. These factors may have contributed to the lack of significant changes observed in certain APG indices, such as the b/a, d/a, and e/a ratios, in our study. More controlled conditions and extended observation periods are required to detect subtle hemodynamic alterations.

No additional changes in APG parameters were observed after moxibustion. This finding suggests that the hemodynamic response detected by APG occurred primarily after acupuncture, with no additional changes observed following moxibustion during the treatment session.

### 4.3. PW Doppler US and the Role of Moxibustion in Vascular Changes

PW Doppler parameters did not change significantly after acupuncture alone. In contrast to the APG findings, acupuncture alone did not produce significant changes in PW Doppler parameters, which reflect regional blood flow in larger vessels [30]. Acupuncture may modulate hemodynamics by reducing whole blood viscosity while promoting microcirculation through increased CYP2C11 expression and microvessel dilation [39]. However, the absence of significant PW Doppler changes may reflect either the limited sensitivity of PW Doppler to microcirculatory changes or an insufficient acupuncture dose or duration to alter temporal artery blood flow.

After combined acupuncture and moxibustion treatment, PW Doppler demonstrated significant increases in PSV, EDV, and MV, together with a decrease in RI. The significant increase in PSV and EDV points to improved arterial blood flow, likely facilitated by enhanced vasodilation and reduced vascular resistance, as reflected by the reduced RI. The increase in MV further underscores this improvement in overall blood flow. These findings are consistent with previous studies suggesting that moxibustion may influence vascular tone and blood flow [18,20]. However, because no local heat control was included, the observed vascular changes cannot be distinguished from the general vasodilatory effects of local heat. These findings suggest that PW Doppler provides complementary information on hemodynamic responses during acupuncture and moxibustion treatment.

### 4.4. Variability in Patient Response

Our findings showed that hemodynamic changes measured by APG and PW Doppler did not consistently correspond with changes in THI scores. For example, Subject 2 showed the greatest reduction in THI (−14) despite no significant changes in APG or PW Doppler metrics, while Subject 3 had marked hemodynamic improvements but a slight increase in THI (+2), indicating worsened tinnitus symptoms. Conversely, Subjects 4, 6, and 8 showed both hemodynamic improvements and tinnitus relief (THI −8, −2, and −4, respectively).

These findings align with previous research, suggesting that tinnitus alleviation is not always directly linked to measurable peripheral vascular changes. Additional factors, such as neural plasticity or central auditory processing, may also contribute to tinnitus symptoms [3]. The results underscore the intricate nature of tinnitus pathophysiology, explaining the variability observed in individual treatment responses. These acute vascular responses may not predict long-term changes in THI scores. Therefore, further studies are needed to determine their clinical relevance to tinnitus symptoms.

### 4.5. Strengths and Limitations

This study is significant as it is one of the first to combine PPG and PW Doppler US for a non-invasive assessment of hemodynamic changes during acupuncture and moxibustion treatment in patients with tinnitus. By addressing the limitations of each diagnostic tool, our findings suggest the potential of APG and PW Doppler US as objective tools for monitoring hemodynamic changes during acupuncture and moxibustion treatment.

Focusing on the temporal artery, rather than the more commonly studied cortical blood flow [22,23,24,25] or carotid artery [10,31,32], represents another key strength. The temporal artery is readily accessible, allowing for direct assessment of peripheral circulation using a practical and non-invasive approach. This approach could reveal vascular patterns or abnormalities that might be overlooked when focusing exclusively on cerebral circulation. Comparison with previous studies of cortical blood flow may provide a more comprehensive understanding of vascular responses to acupuncture and moxibustion.

However, the small sample size of 10 participants limits the statistical power and generalizability of the findings, and the results should therefore be interpreted as preliminary. The heterogeneous causes of tinnitus may also have influenced the hemodynamic responses, and this could not be adequately controlled for because of the small sample size. In addition, multiple comparisons across hemodynamic parameters may have increased the risk of Type I error. Because multiple local and distal acupoints were used, the observed temporal artery changes cannot be attributed specifically to SI19 and may reflect both local and systemic effects. The retrospective design, relying on pre-existing medical records, poses a risk of information bias due to potential inaccuracies or incomplete data. The lack of a control group limits assessment of placebo or nonspecific effects. Potential confounding factors could not be fully controlled. Moreover, the single-center setting and four-month study period may limit the generalizability of the findings and assessment of long-term effects. Additionally, the focus on immediate hemodynamic changes limits our understanding of longer-term hemodynamic responses. Lastly, the diagnostic tools used have inherent limitations, as both PPG and PW Doppler are susceptible to external factors such as motion artifacts and sensor placement, which may have introduced variability in the measurements.

### 4.6. Suggestions for Future Research

Future research should include larger randomized controlled trials to confirm these preliminary findings and investigate both immediate and long-term changes in APG and PW Doppler parameters, together with changes in tinnitus symptoms. To achieve this, future studies should include longer treatment periods, larger sample sizes, and additional non-invasive hemodynamic assessment methods. It is also essential to explore the neurophysiological effects of these interventions, using objective neurophysiological measures related to central auditory pathways and neural plasticity. Future studies should also examine whether patient-specific factors, such as baseline cardiovascular health or tinnitus severity, influence treatment outcomes. Finally, future research should investigate how hemodynamic responses vary according to the selection of acupoints used in tinnitus management.

## 5. Conclusions

This study demonstrated that APG and PW Doppler can objectively characterize hemodynamic changes during acupuncture and moxibustion treatment in patients with subjective tinnitus. APG detected changes in arterial pulse wave characteristics after acupuncture, whereas PW Doppler identified additional changes in superficial temporal artery blood flow following combined acupuncture and moxibustion treatment. Together, APG and PW Doppler provided complementary information on acute hemodynamic responses during treatment. However, these hemodynamic changes did not consistently correspond with changes in tinnitus symptoms. The variability in individual treatment responses underscores the complex nature of tinnitus pathophysiology and highlights the need for individualized assessment. Further research with larger sample sizes, longer follow-up periods, and more advanced hemodynamic and neurophysiological assessments is essential to validate these findings and refine the use of acupuncture and moxibustion in tinnitus care.

## Figures and Tables

**Table 1:** Characteristics of Tinnitus Patients (*N* = 10).

Characteristics (N = 10)	Mean (SD ^1^) or Number (%)
Male	6 (60.0%)
Age (years)	41.8 (±18.4)
Onset (months)	12.1 (±11.5)
Tinnitus site	
Bilateral	2 (20.0%)
Unilateral	8 (80.0%)
Cause of onset	
Stress	1 (10.0%)
Traumatic	1 (10.0%)
Sudden hearing loss	2 (20.0%)
Otitis media, Meniere’s disease, flu	1 (10.0%)
BPPV ^2^	1 (10.0%)
Unknown cause	4 (40.0%)
Number of outpatient treatments	6.9 (±3.9)
THI ^3^ at the first outpatient visit	26.8 (±12.9)
Symptoms related to ear	
Aural fullness	7 (70.0%)
Hearing loss	4 (40.0%)
Otalgia	4 (40.0%)
Vertigo	2 (20.0%)

^1^ SD, standard deviation; ^2^ BPPV, benign paroxysmal positional vertigo; ^3^ THI, tinnitus handicap inventory.

**Table 2:** Mean Values and Standard Deviations for the ‘b/a’, ‘c/a’, ‘d/a’, and ‘e/a’ Ratios Across Three Groups: Base (Before Treatment), AT (After Acupuncture), and AT + MT (After Acupuncture Combined with Moxibustion).

	b/a	c/a	d/a	e/a
Base	−0.850 (±0.168)	−0.179 (±0.158)	−0.241 (±0.151)	0.192 (±0.069)
AT	−0.833 (±0.212)	−0.126 (±0.156)	−0.236 (±0.178)	0.198 (±0.073)
AT + MT	−0.840 (±0.208)	−0.136 (±0.178)	−0.250 (±0.170)	0.199 (±0.048)

Data are mean (±SD).

**Table 3:** Statistical Analysis of the ‘b/a’ and ‘c/a’ Ratios Across Three Groups: Base (Before Treatment), AT (After Acupuncture), and AT + MT (After Acupuncture Combined with Moxibustion).

	Base vs. AT		Base vs. AT + MT		AT vs. AT + MT	
	b/a	c/a	b/a	c/a	b/a	c/a
Effect	0.017 (±0.021)	0.053 (±0.017)	0.010 (±0.021)	0.043 (±0.018)	−0.007 (±0.022)	−0.010 (±0.019)
*p*-value	0.425	0.002 **	0.649	0.020 *	0.739	0.598
Group variance	0.035 (±0.186)	0.021 (±0.140)	0.035 (±0.189)	0.026 (±0.158)	0.044 (±0.224)	0.026 (±0.160)

Data are mean (±SE). ** *p*-value < 0.01; * *p*-value < 0.05.

**Table 4:** Statistical Analysis of the ‘d/a’ and ‘e/a’ Ratios Across Three Groups: Base (Before Treatment), AT (After Acupuncture), and AT + MT (After Acupuncture Combined with Moxibustion).

	Base vs. AT		Base vs. AT + MT		AT vs. AT + MT	
	d/a	e/a	d/a	e/a	d/a	e/a
Effect	0.005 (±0.020)	0.006 (±0.011)	−0.009 (±0.018)	0.007 (±0.010)	−0.014 (±0.016)	0.001 (±0.009)
*p*-value	0.800	0.575	0.624	0.458	0.386	0.920
Group variance	0.026 (±0.149)	0.003 (±0.031)	0.026 (±0.167)	0.002 (±0.023)	0.034 (±0.240)	0.002 (±0.029)

Data are mean (±SE).

**Table 5:** Mean Values and Standard Deviations for the PW Doppler Ultrasound Parameters Across Three Groups: Base (Before Treatment), AT (After Acupuncture), and AT + MT (After Acupuncture Combined with Moxibustion).

	PSV ^1^	EDV ^2^	RI ^3^	MV ^4^
Base	31.248 (±12.913)	5.812 (±2.832)	0.802 (±0.084)	18.171 (±7.675)
AT	32.539 (±11.883)	6.547 (±3.221)	0.786 (±0.095)	19.566 (±6.837)
AT + MT	35.609 (±11.403)	7.620 (±3.141)	0.775 (±0.089)	21.622 (±6.518)

Data are mean (±SD). ^1^ PSV, Peak Systolic Velocity; ^2^ EDV, End Diastolic Velocity; ^3^ RI, Resistive Index; ^4^ MV, Mean flow Velocity.

**Table 6:** Statistical Analysis of Peak Systolic Velocity (PSV) and End Diastolic Velocity (EDV) Across Three Groups: Base (Before Treatment), AT (After Acupuncture), and AT + MT (After Acupuncture Combined with Moxibustion).

	Base vs. AT		Base vs. AT + MT		AT vs. AT + MT	
	PSV	EDV	PSV	EDV	PSV	EDV
Effect	1.291 (±2.107)	0.734 (±0.559)	4.360 (±1.870)	1.807 (±0.537)	3.069 (±2.001)	1.073 (±0.584)
*p*-value	0.540	0.189	0.020 *	0.001 **	0.125	0.066
Group variance	57.578 (±3.239)	2.709 (±0.648)	75.327 (±4.492)	3.419 (±0.833)	50.857 (±3.046)	3.018 (±0.681)

Data are mean (±SE). ** *p*-value < 0.01; * *p*-value < 0.05.

**Table 7:** Statistical Analysis of the Resistive Index (RI) and Mean Flow Velocity (MV) Across Three Groups: Base (Before Treatment), AT (After Acupuncture), and AT + MT (After Acupuncture Combined with Moxibustion).

	Base vs. AT		Base vs. AT + MT		AT vs. AT + MT	
	RI	MV	RI	MV	RI	MV
Effect	−0.016 (±0.011)	1.395 (±1.270)	−0.027 (±0.011)	3.451 (±1.140)	−0.011 (±0.012)	2.055 (±1.199)
*p*-value	0.157	0.272	0.014 *	0.002 **	0.332	0.086
Group variance	0.006 (±0.054)	18.257 (±1.762)	0.005 (±0.050)	24.263 (±2.443)	0.006 (±0.055)	14.181 (±1.495)

Data are mean (±SE). ** *p*-value < 0.01; * *p*-value < 0.05.

**Table 8:** Pre- and Post-Treatment Differences in the Tinnitus Handicap Inventory (THI) and Hemodynamic Parameters for Individual Patients.

Subject	ΔTHI ^1^	Δb/a	Δc/a	Δd/a	Δe/a	ΔPSV ^2^	ΔEDV ^3^	ΔRI ^4^	ΔMV ^5^
1	−4	0.920	0.090	0.467	0.291	0.338	0.159	0.214	0.239
2	−14	0.322	0.125	1.000	0.728	0.893	0.379	0.321	0.798
3	2	0.035 *	0.324	0.001 **	0.840	0.045 *	0.004 **	0.250	0.018 *
4	−8	0.801	0.048 *	0.343	0.153	0.125	0.165	0.035 *	0.125
5	−6	0.413	0.853	0.201	0.803	0.920	0.345	0.120	0.729
6	−2	0.790	0.218	0.289	0.797	0.137	0.020 *	0.096	0.089
7	−4	0.793	0.936	0.900	0.169	0.823	0.796	0.562	0.921
8	−4	0.804	0.043 *	0.509	0.728	0.383	0.217	0.309	0.223

^1^ ΔTHI, THI score at the last outpatient visit—THI score at the first outpatient visit; ^2^ PSV, Peak Systolic Velocity; ^3^ EDV, End Diastolic Velocity; ^4^ RI, Resistive Index; ^5^ MV, Mean Flow Velocity; ** *p*-value < 0.01; * *p*-value < 0.05. *p*-values were calculated from repeated pre- and post-treatment measurements across multiple treatment sessions for each patient.

## Data Availability

The raw data supporting the conclusions of this article are available from the corresponding author upon reasonable request, without undue reservation.

## Supplementary Materials

The following supporting information can be downloaded at: https://www.mdpi.com/article/10.3390/audiolres16050130/s1, Table S1: STROBE Statement—Checklist of items that should be included in reports of observational studies.

## Abbreviations

The following abbreviations are used in this manuscript:

PG	Photoplethysmogram
PW Doppler	Pulsed-Wave Doppler
APG	Acceleration Photoplethysmogram
PSV	Peak Systolic Velocity
EDV	End Diastolic Velocity
RI	Resistive Index
MV	Mean Flow Velocity
THI	Tinnitus Handicap Inventory
fNIRS	Functional Near-Infrared Spectroscopy
fMRI	Functional Magnetic Resonance Imaging
Tc-ECD SPECT	Technetium-99m Ethyl Cysteinate Dimer Single Photon Emission Computed Tomography
US	Ultrasound
IRB	Institutional Review Board
RMSK	Registered in Musculoskeletal
APCA	Alliance for Physician Certification & Advancement
SD	Standard Deviations
SE	Standard Errors
LMMs	Linear Mixed-Effects Models
BPPV	Benign Paroxysmal Positional Vertigo
NO	Nitric Oxide
ET-1	Endothelin-1
