# Supplementary material for: Hemodynamic Changes Following Acupuncture and Moxibustion in Patients with Subjective Tinnitus: An Acceleration Photoplethysmography and Pulsed-Wave Doppler Ultrasound Study
Source: Audiol Res. 2026 Sep 2;16(5):130. doi: 10.3390/audiolres16050130 (PMC13600091; doi:10.3390/audiolres16050130)
Supplement: Supplementary file 1 [file audiolres-16-00130-s001.zip › audiolres-4468273-supplementary.pdf]

STROBE Statement—Checklist of items that should be included in reports of observational studies

Manuscript: Hemodynamic changes following acupuncture and moxibustion in patients with subjective tinnitus: An acceleration photoplethysmography and pulsed-wave Doppler ultrasound study

Section/Topic	Item No.	Recommendation	Reported in manuscript	Page/line
Title and abstract	1	(a) Indicate the study's design with a commonly used term in the title or the abstract	Abstract identifies the study as a retrospective review.	Page 1, lines 11–25
Title and abstract	1	(b) Provide in the abstract an informative and balanced summary of what was done and what was found	The abstract summarizes participants, measurements, main hemodynamic findings, and THI findings.	Page 1, lines 11–25
Introduction – Background/rationale	2	Explain the scientific background and rationale for the investigation being reported	Background on tinnitus, vascular factors, acupuncture/moxibustion, and non-invasive hemodynamic assessment is provided.	Pages 1–2, lines 29–86
Introduction – Objectives	3	State specific objectives, including any prespecified hypotheses	The objective is to investigate hemodynamic changes during combined acupuncture and moxibustion using APG and PW Doppler, including individual variability.	Page 3, lines 87–92
Methods – Study design	4	Present key elements of study design early in the paper	The study is described as a retrospective chart review; APG and PW Doppler were routine clinical assessments retrospectively reviewed.	Page 3, lines 94–106
Methods – Setting	5	Describe the setting, locations, and relevant dates, including periods of recruitment, exposure, follow-up, and data collection	Hospital, outpatient department, location, and April–July 2024 study period are reported.	Page 3, lines 95–116
Methods – Participants	6	Give the eligibility criteria, and the sources and methods of selection of participants	Inclusion and exclusion criteria and availability of APG/PW Doppler data are described.	Pages 3–4, lines 111–131
Methods – Variables	7	Clearly define all outcomes, exposures, predictors, potential confounders, and effect modifiers. Give diagnostic criteria, if applicable	Tinnitus characteristics, THI, APG variables, and PW Doppler variables are defined; subjective non-pulsatile tinnitus is specified in the inclusion criteria.	Pages 3 and 7, lines 119–122 and 215–244
Methods – Data sources/measurement	8	For each variable of interest, give sources of data and details of methods of assessment (measurement). Describe comparability of assessment methods if there is more than one group	Electronic medical records, PPG protocol, PW Doppler protocol, measurement timing, devices, and measured Doppler parameters are described.	Pages 3–5 and 7, lines 112–116, 132–169, and 225–244
Methods – Bias	9	Describe any efforts to address potential sources of bias	Standardized measurement conditions are described; potential information bias, confounding, lack of control, and measurement variability are acknowledged as limitations.	Pages 4 and 16, lines 133–139 and 494–510
Methods – Study size	10	Explain how the study size was arrived at	The retrospective review included patients with available APG and PW Doppler data during April–July 2024; no formal sample-size calculation is reported. The small sample size is acknowledged as a limitation.	Pages 3 and 16, lines 95–98, 111–112, and 494–498
Methods – Quantitative variables	11	Explain how quantitative variables were handled in the analyses. If applicable, describe which groupings were chosen and why	APG and PW Doppler variables are defined, and continuous variables are summarized using means with SD or SE.	Pages 7–8, lines 225–266
Methods – Statistical methods	12	(a) Describe all statistical methods, including those used to control for confounding	LMMs, Shapiro–Wilk tests, paired t-tests, and Wilcoxon signed-rank tests are described.	Page 8, lines 249–266
Methods – Statistical methods	12	(b) Describe any methods used to examine subgroups and interactions	Individual analyses were limited to patients with at least three treatment sessions for paired comparisons.	Page 8, lines 259–266
Methods – Statistical methods	12	(c) Explain how missing data were addressed	No specific missing-data analysis or imputation method is reported; inclusion required available APG and PW Doppler data.	Page 3, lines 111–122
Methods – Statistical methods	12	(d) If applicable, explain how loss to follow-up was addressed	Not applicable to the retrospective repeated-measure analysis as reported; individual analysis was restricted to patients with at least three sessions.	Page 8, lines 259–266
Methods – Statistical methods	12	(e) Describe any sensitivity analyses	No sensitivity analysis is reported.	Not reported
Results – Participants	13	(a) Report numbers of individuals at each stage of study	The flow diagram reports the retrospective chart review process and final analysis of 10 patients.	Page 8, lines 269–282 (Figure 8)
Results – Participants	13	(b) Give reasons for non-participation at each stage	Participant selection is presented in Figure 8; individual analysis was limited to eight subjects with at least three outpatient visits.	Pages 8 and 13, lines 269–282 and 371–386
Results – Participants	13	(c) Consider use of a flow diagram	A flow diagram is provided.	Page 8, lines 269–282 (Figure 8)
Results – Descriptive data	14	(a) Give characteristics of study participants and information on exposures and potential confounders	Age, sex, tinnitus duration/site/cause, number of treatments, THI, and ear-related symptoms are reported.	Pages 8–9, lines 270–288 (Table 1)
Results – Descriptive data	14	(b) Indicate number of participants with missing data for each variable of interest	Not separately reported.	Not reported
Results – Outcome data	15	Report numbers of outcome events or summary measures	APG, PW Doppler, and THI-related outcome data are reported in Tables 2–8 and Figures 9–11.	Pages 9–13, lines 289–396
Results – Main results	16	(a) Give unadjusted estimates and, if applicable, confounder-adjusted estimates and their precision	LMM effect estimates, SEs, P-values, and group variance are reported for APG and PW Doppler parameters.	Pages 9–12, lines 289–369
Results – Main results	16	(b) Report category boundaries when continuous variables were categorized	Not applicable; the principal hemodynamic variables were analyzed as continuous variables.	Pages 7–8, lines 225–266
Results – Main results	16	(c) If relevant, consider translating estimates of relative risk into absolute risk for a meaningful time period	Not applicable; no relative-risk estimates were used.	Not applicable
Results – Other analyses	17	Report other analyses done—e.g., analyses of subgroups and interactions, and sensitivity analyses	Individual patient analyses of THI, APG, and PW Doppler changes are reported for eight subjects with at least three visits.	Page 13, lines 370–396
Discussion – Key results	18	Summarise key results with reference to study objectives	The Discussion opens by summarizing the study's assessment of hemodynamic changes during acupuncture and moxibustion.	Page 14, lines 398–400; Pages 14–16, lines 401–485
Discussion – Limitations	19	Discuss limitations of the study, taking into account sources of potential bias or imprecision	Small sample size, heterogeneity, multiple comparisons, local/systemic effects, retrospective information bias, lack of control, confounding, single-center setting, short study period, lack of long-term assessment, and measurement limitations are acknowledged.	Page 16, lines 494–510
Discussion – Interpretation	20	Give a cautious overall interpretation of results considering objectives, limitations, multiplicity of analyses, results from similar studies, and other relevant evidence	The Discussion interprets APG and PW Doppler findings cautiously, addresses heat effects and variability, and calls for further research.	Pages 14–16, lines 398–522
Discussion – Generalisability	21	Discuss the generalisability (external validity) of the study results	Limited generalizability due to the small sample, single-center setting, and four-month study period is explicitly acknowledged.	Page 16, lines 494–510
Other information – Funding	22	Give the source of funding and the role of the funders for the present study and, if applicable, for the original study on which the present article is based	The manuscript states that the research received no external funding.	Page 17, line 543

Note: Page and line references correspond to Audiology research_260821(2).pdf. "Not reported" indicates that the revised manuscript does not explicitly provide the requested STROBE information.
